# Comparison of the Microbiota of Older Adults Living in Nursing Homes and the Community

**DOI:** 10.1128/mSphere.00210-17

**Published:** 2017-09-13

**Authors:** Mary-Claire Roghmann, Alison D. Lydecker, Lauren Hittle, Robert T. DeBoy, Rebecca G. Nowak, J. Kristie Johnson, Emmanuel F. Mongodin

**Affiliations:** a Geriatrics Research Education and Clinical Center, VA Maryland Health Care System, Baltimore, Maryland, USA; b Department of Epidemiology and Public Health, University of Maryland School of Medicine, Baltimore, Maryland, USA; c Department of Microbiology and Immunology and Institute for Genome Sciences, University of Maryland School of Medicine, Baltimore, Maryland, USA; d Department of Pathology, University of Maryland School of Medicine, Baltimore, Maryland, USA; Carnegie Mellon University

**Keywords:** microbiota, *Staphylococcus aureus*, Gram-negative bacteria

## Abstract

Our objective for this study was to characterize the microbial communities of the anterior nares (nose), posterior pharynx (throat), and skin of the femoral and subclavian areas in older adults from nursing homes and the community. Older adults (≥65 years) without antibiotic use for the past 3 months were recruited from nursing homes (NH; *n* = 16) and from the community (CB; *n* = 51). Specimens were taken from nose, throat, and skin sites for culture and bacterial profiling using 16S rRNA gene sequencing. We found that pathogenic Gram-negative rod (GNR) colonization on the femoral skin was higher in NH participants than CB participants; otherwise, there were no differences in GNR colonization at other body sites or in *Staphylococcus aureus* colonization at any body site. Bacterial community profiling demonstrated that the operational taxonomic unit compositions of the different body sites were similar between NH and CB participants, but the analysis identified differences in relative abundance levels. *Streptococcus* spp. were more abundant and *Prevotella* spp. were less abundant in the throats of NH participants than in throats of CB participants. *Proteus*, *Escherichia coli*, and *Enterococcus* were more abundant in NH participants on the femoral skin. We found a pattern of decreased abundance of specific *Proteobacteria* in NH participants at the anterior nares and at both skin sites. We concluded that bacterial communities were largely similar in diversity and composition within body sites between older adults without recent antibiotic use from NH compared to those from the community. Our findings support the rationale for improved hygiene in NH residents to reduce the transmission risk of antibiotic-resistant bacteria, such as *Enterococcus* spp. or *Enterobacteriaceae*.

**IMPORTANCE** The nose, throat, and skin over the subclavian and femoral veins are the body sites which harbor the bacteria which most commonly cause health care-associated infection. We assessed the effect of nursing home residence on the microbiota of these body sites in older adults. We found that the microbiota composition of the different body sites was similar between nursing home and community participants, but we identified differences in relative abundance levels. We found remarkable similarities in the bacterial communities of different body sites in older adults who lived in nursing homes compared to those in the community among people who had not been on antibiotics for the past 3 months. We also found that the femoral skin microbiota had evidence of stool contamination in the nursing home residents, providing a rationale for improved skin hygiene. Taken together, it appears that the health care environment does not alter the microbiota to the extent that antibiotics do.

## Abstract (precis)

The nose, throat, and skin over the subclavian and femoral veins are the body sites which harbor the bacteria which most commonly cause health care-associated infection. We assessed the effect of nursing home residence on the microbiota of these body sites in older adults. We found that the microbiota composition of the different body sites was similar between nursing home and community participants, but we identified differences in relative abundance levels. We found remarkable similarities in the bacterial communities of different body sites in older adults who lived in nursing homes compared to those in the community among people who had not been on antibiotics for the past 3 months. We also found that the femoral skin microbiota had evidence of stool contamination in the nursing home residents, providing a rationale for improved skin hygiene. Taken together, it appears that the health care environment does not alter the microbiota to the extent that antibiotics do.

## INTRODUCTION

The microbiota of older adults living in nursing homes has long been felt to be different than that of older adults living independently in the community. Johansen and colleagues showed that the prevalence of Gram-negative bacteria in the oropharynx is low in physiologically normal subjects even when exposed to the hospital environment (1, 2); however, the prevalence of Gram-negative bacteria increases substantially with the onset of illness. Valenti and colleagues showed that oropharyngeal colonization with *Klebsiella* spp., *Escherichia coli*, and *Enterobacter* spp. increased with level of care in older adult populations, from healthy older adult volunteers to hospitalized patients and debilitated nursing home residents (3). Nursing home residents are well known to have a higher prevalence of colonization with multidrug-resistant organisms, including Gram-negative bacteria (4–6). This prior work was based on culture-based methods which focused specifically on pathogenic Gram-negative bacteria. Using DNA sequence-based methods, we were able to assess the entire microbiota without the bias of what microorganisms were cultivable.

In this study, we assessed the effect of nursing home residence on the microbiota of the body sites that harbor the bacteria which most commonly cause health care-associated infections: the anterior nares, posterior pharynx, and the skin over the subclavian and femoral veins. We conducted a cross-sectional study of older adults from two settings: nursing homes and the community. Our primary objective was to characterize the bacterial communities in the nose, throat, and skin sites in older adults and to compare them based on the study participant’s living environment. We hypothesized that culture positivity and bacterial profiles at each body site would differ by setting.

## RESULTS

### Study population.

We screened 62 older adults who lived in the community and enrolled 51 participants. We screened 24 older adults who lived in nursing homes and enrolled 16 participants. Baseline characteristics of our participants are shown in Table 1. The nursing home (NH) participants were older, more likely to be male, have diabetes, and have an indwelling medical device compared to the community participants. The community-based (CB) participants were more likely to have bathed or showered in the last 12 h. No participants smoked cigarettes.

**TABLE 1:** Baseline characteristics and bacterial colonization by living environment

Characteristic^a^	No. (%) with characteristic in study group			*P* value
	Overall population (*n* = 67)	Nursing home (*n* = 16)	Community (*n* = 51)	
Age (median yrs [IQR])	72 (69, 79)	78 (70, 89)	71 (68, 76)	0.04
Male	34 (51)	13 (81)	21 (41)	<0.01
Race or ethnicity				0.31
Asian	2 (3)	0 (0)	2 (4)	
Black or African American	15 (23)	6 (38)	9 (18)	
White	46 (71)	10 (63)	36 (73)	
Multiracial	2 (3)	0 (0)	2 (4)	
Body mass index (median [IQR])	27 (25, 31)	29 (26, 34)	27 (25, 30)	0.25
Diabetes	7 (11)	7 (47)	0 (0)	<0.01
Indwelling medical device	2 (3)	2 (13)	0 (0)	0.05
Bathed or showered in past 12 h	37 (56)	3 (20)	34 (67)	<0.01
Colonized with *Staphylococcus aureus*				
Any body site	20 (30)	4 (25)	16 (31)	0.76
Nose	14 (22)	3 (20)	11 (22)	1.00
MRSA	4 (6)	1 (7)	3 (6)	1.00
MSSA	10 (15)	2 (13)	8 (16)	1.00
Throat	11 (17)	2 (15)	9 (18)	1.00
MRSA	2 (3)	0 (0)	3 (4)	1.00
MSSA	9 (14)	2 (15)	7 (14)	1.00
Subclavian skin	4 (7)	0 (0)	4 (9)	0.57
MRSA	2 (3)	0 (0)	2 (4)	1.00
MSSA	2 (3)	0 (0)	2 (4)	1.00
Femoral skin	3 (5)	1 (6)	2 (4)	1.00
MRSA	2 (3)	1 (6)	1 (2)	0.44
MSSA	1 (2)	0 (0)	1 (2)	1.00
Colonized with pathogenic GNR				
Any body site	29 (43)	9 (56)	20 (39)	0.23
Nose	19 (29)	6 (40)	13 (26)	0.30
*Pseudomonas aeruginosa*	1 (2)	1 (1)	0 (0)	0.23
*Acinetobacter* spp.	2 (3)	1 (7)	1 (2)	0.41
* Enterobacteriaceae*	18 (28)	6 (40)	12 (24)	0.23
Throat	16 (25)	3 (23)	13 (25)	1.00
*Pseudomonas aeruginosa*	1 (2)	1 (8)	0 (0)	0.20
*Acinetobacter* spp.	0 (0)	0 (0)	0 (0)	
* Enterobacteriaceae*	16 (25)	3 (23)	13 (25)	1.00
Subclavian skin	4 (7)	2 (15)	2 (4)	0.20
*Pseudomonas aeruginosa*	1 (2)	1 (8)	0 (0)	0.22
*Acinetobacter* spp.	0 (0)	0 (0)	0 (0)	
* Enterobacteriaceae*	4 (7)	2 (15)	2 (4)	0.20
Femoral skin	12 (18)	7 (44)	5 (10)	<0.01
*Pseudomonas aeruginosa*	2 (3)	2 (13)	0 (0)	0.06
*Acinetobacter* spp.	1 (2)	1 (6)	0 (0)	0.25
* Enterobacteriaceae*	11 (17)	6 (38)	5 (10)	0.02

a For GNR data, participants could be colonized with more than one *Enterobacteriaceae* species. Analysis was performed per participant. The three most common organisms from the *Enterobacteriaceae* family found at each body site were *Escherichia coli*, *Klebsiella pneumoniae*, and *Proteus mirabilis*. IQR, interquartile range; MRSA, methicillin-resistant *S. aureus*; MSSA, methicillin-sensitive *S. aureus*.

### Colonization with *S. aureus* and pathogenic GNR by culture.

Participant *Staphylococcus aureus* and pathogenic Gram-negative rod (GNR) bacterial colonization by body site and living environment are summarized in Table 1. There were no significant differences in colonization with either group of bacteria with the exception of pathogenic GNR, which were significantly more common in the femoral area of NH participants than CB participants. This difference was due to colonization with *Enterobacteriaceae* (38% versus 10%; *P* < 0.01, Fisher’s exact test) and *Pseudomonas aeruginosa* (13% versus 0%; *P* = 0.06, Fisher’s exact test).

### 16S rRNA gene sequencing data set.

Bacterial community profiling using 16S rRNA gene sequencing was performed on a total of 268 samples. Of these 268 samples, 14 samples with a Good’s sequence coverage index of <90% were removed from further downstream analyses, for a final set of 254 samples analyzed. The final sequencing data set contained a total of 16,383,325 16S rRNA gene sequences from 254 samples (64,501 sequences were obtained on average per sample, with a range from 500 to 215,348 sequences), representing 6,269 unique operational taxonomic units (OTUs) at a 97% similarity cutoff across all samples.

### Alpha diversity.

**FIG 1:**
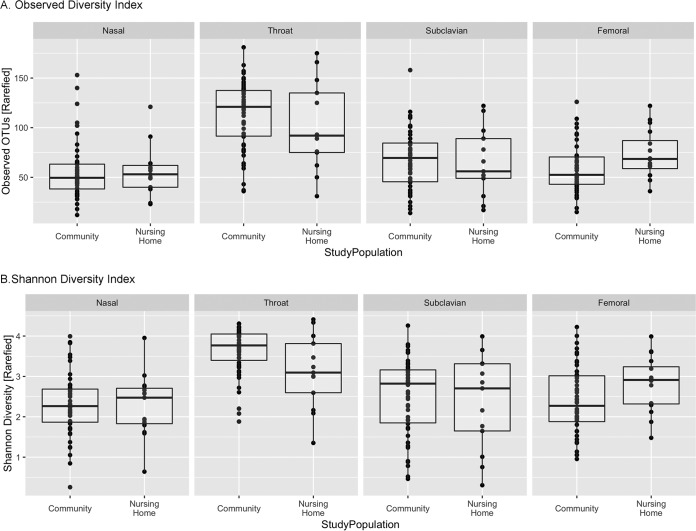
Alpha diversity analyses of the samples for comparison of body sites between the two study populations at a sequencing depth of 500 sequences per sample. (A) Observed diversity index; (B) Shannon diversity index.

Alpha diversity, a method to quantitate intrasample diversity, was calculated using the Phyloseq package in R and was reported by using the observed diversity index (total number of OTUs, a measure of community richness) and Shannon diversity index. Because differences in sequence coverage, even close to sequence saturation, can have a significant impact on alpha diversity measures, we calculated the observed diversity index and Shannon diversity index (Fig. 1) on rarefied data. As expected, the median richness and Shannon diversity index varied by body site, with the highest level at the throat. The median richness and Shannon diversity index were similar between the two study populations at the nose and subclavian skin. The median richness (92 versus 121; *P* = 0.30, Wilcoxon rank sum) and Shannon diversity index (3.10 versus 3.77; *P* = 0.06) of the throat were lower in the nursing home participants. In contrast, the median richness (69 versus 53; *P* = 0.03) and Shannon diversity index (2.91 versus 2.27; *P* = 0.08) of the femoral skin site were higher in the nursing home participants. Alpha diversity comparisons on rarefied and nonrarefied data sets yielded the same results (see Fig. S1 in the supplemental material).

10.1128/mSphere.00210-17.1 FIG S1  Alpha diversity analyses of the samples, showing results for comparisons of body sites between study populations (unrarefied and rarefied data; sequencing depth of 500 sequences per sample). (A) Observed diversity index; (B) Shannon diversity index. Download FIG S1, TIF file, 0.2 MB. Copyright © 2017 Roghmann et al. 2017 Roghmann et al. This content is distributed under the terms of the Creative Commons Attribution 4.0 International license.

### Beta diversity.

**FIG 2:**
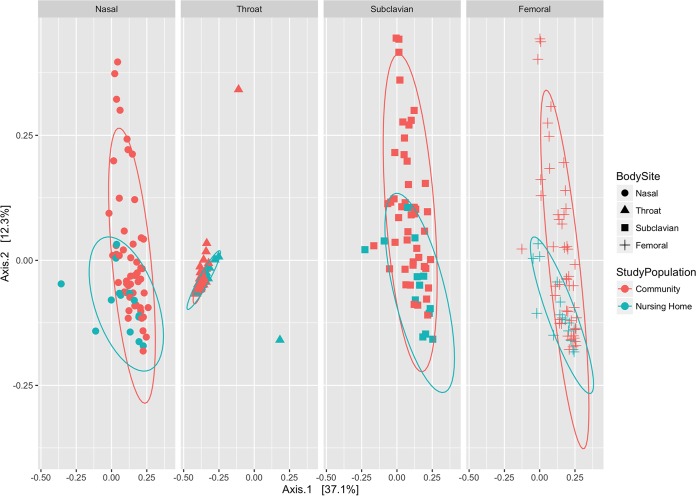
PCoA results for beta diversity metrics between study populations by body site, showing distances from the Jensen-Shannon divergence. The eight ellipses represent 95% confidence intervals for clustered specimens from older adults from the nursing home and community study populations at each of the four body sites. ANOSIM test of significance results: nasal, *R* =0.1463, *P* = 0.04; throat, *R* = 0.3401, *P* = 0.003; subclavian skin, *R* = 0.04798, *P* = 0.297; femoral skin, *R* = 0.1815, *P* = 0.026.

Comparison of intersample diversity was characterized using beta diversity analyses based on the Jensen-Shannon divergence. A comparison of bacterial community structures between nursing home and community participants by body site showed that samples of nursing home participants clustered with overlap to the community participants (Fig. 2). However, an ANOSIM (ANalysis of SIMilarity) test of significance, applied to the measurements of ordination distances from Jensen-Shannon divergence, suggested that the clusters that were observed for nursing home and community-based study populations had significant differences at three body sites: nasal (*R* = 0.1463, *P* = 0.04), throat (*R* = 0.3401, *P* = 0.003), and femoral skin (*R* = 0.1815, *P* = 0.026). Although no significant difference was detected at the subclavian skin (*R* = 0.04798, *P* = 0.297), this might have been due to the wider spread of data points within the CB compared to the NH samples. These observations were confirmed by similar clustering results when other beta diversity measures (weighted and unweighted UniFrac) were employed (Fig. S2). Of note, the samples from community and nursing home participants overlapped completely with unweighted UniFrac analysis (which scores the presence or absence of taxa), but not with weighted UniFrac analysis (which scores the relative abundance of taxa). These observations are consistent with the view that the two study populations are mostly similar overall in bacterial composition but differ in relative abundance of some taxa at some body sites. No collected metadata variables could explain the difference in OTU composition at these body sites, including age, sex, race/ethnicity, body mass index (BMI), diabetes, or presence of a medical device at enrollment or recent shower/bath (Fig. S3).

10.1128/mSphere.00210-17.2 FIG S2  PCoA results for beta diversity metrics between study populations by body site, showing distances determined from weighted and unweighted UniFrac analyses. The eight ellipses represent 95% confidence intervals for clustered specimens from older adults from the nursing home and community study populations at each of the four body sites. Download FIG S2, TIF file, 2 MB. Copyright © 2017 Roghmann et al. 2017 Roghmann et al. This content is distributed under the terms of the Creative Commons Attribution 4.0 International license.

10.1128/mSphere.00210-17.3 FIG S3  PCoA results for beta diversity metrics between study populations by body site, showing distances determined from the Jensen-Shannon divergence based on collected metadata, including age, sex, race, or ethnicity, BMI, diabetes, and presence of a medical device at enrollment or recent shower or bath. Download FIG S3, TIF file, 0.9 MB. Copyright © 2017 Roghmann et al. 2017 Roghmann et al. This content is distributed under the terms of the Creative Commons Attribution 4.0 International license.

### Microbiome composition by body site and study population.

**FIG 3:**
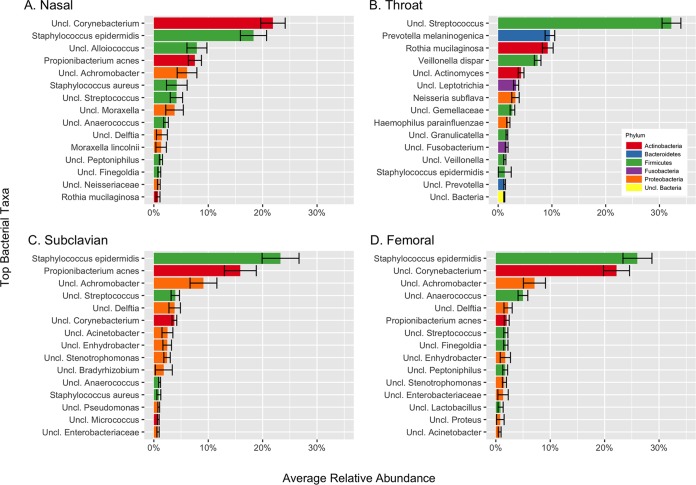
Distribution of the top 15 bacterial taxa at the lowest taxonomic classification for each body site. Error bars for each taxa show standard errors, calculated across all of the patients and all of the samples for nose (A), throat (B), subclavian skin (C), and femoral skin (D).

The distributions of the top 15 bacterial taxa at the lowest taxonomic classification for each body site are shown in Fig. 3. *Corynebacterium* and *Staphylococcus epidermidis* were the most abundant bacterial taxa in the anterior nares. *Streptococcus*, *Prevotella melaninogenica*, *Rothia mucilaginosa*, and *Veillonella dispar* were the most abundant bacterial taxa in the throat. *S. epidermidis*, *Propionibacterium acnes*, and *Achromobacter* were the most abundant bacterial taxa in the subclavian skin. *S. epidermidis*, *Corynebacterium*, and *Achromobacter* were the most abundant bacterial taxa in the femoral skin.

**FIG 4:**
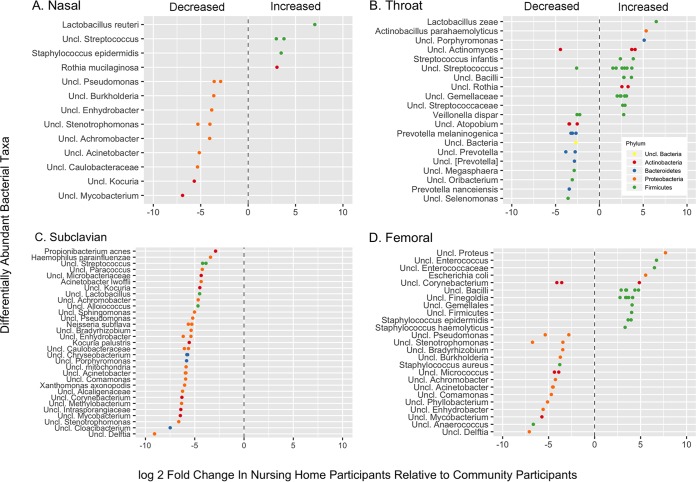
Differentially abundant bacteria in 4 body sites in older adults in nursing homes (*n* = 16) relative to abundance levels in older adults in the community (*n* = 51), calculated using generalized linear models of abundance based on a negative binomial distribution (29). (A) Nose (*n* = 15 NH, *n* = 50 CB); (B) throat (*n* = 13 NH, *n* = 51 CB); (C) subclavian skin (*n* = 13 NH, *n* = 47 CB); (D) femoral skin (*n* = 16 NH, *n* = 49 CB).

We identified a number of OTUs that were significantly differentially abundant between nursing home and community participants (Fig. 4). In the anterior nares, *Lactobacillus reuteri*, two unclassified *Streptococcus* isolates, *S. epidermidis* and an *R. mucilaginosa*, were more abundant in the nursing home participants. In contrast, *Proteobacteria*, including unclassified *Pseudomonas*, *Burkholderia*, *Enhydrobacter*, *Stenotrophomonas*, *Achromobacter*, *Acinetobacter*, and *Caulobacteraceae* were more abundant in the community participants. In the throat, *Firmicutes*, such as *Lactobacillus zeae*, *Streptococcus infantis*, unclassified *Streptococcus*, and unclassified *Gemellae* were more common in the nursing home participants. *Bacteroidetes*, such as *Prevotella*, were relatively more abundant in the community participants. On the skin above the subclavian vein, numerous OTUs, predominantly *Proteobacteria*, were decreased in nursing home participants compared to community participants. On the skin above the femoral vein, *Firmicutes*, including coagulase-negative *Staphylococcus* and *Finegoldia*, were more abundant in nursing home participants, as were *Proteus*, *E. coli*, and enterococci. Other *Proteobacteria*, such as *Pseudomonas*, *Burkholderia*, *Enhydrobacter*, *Stenotrophomonas*, *Achromobacter*, and *Acinetobacter*, were less abundant in the nursing home participants.

To assess whether recent bath or shower was responsible for the difference in *Proteus*, *E. coli*, and *Enterococcus* isolation, we looked for differentially abundant taxa in those participants with and without a recent bath or shower (Fig. S4). Participants who had showered or bathed were less likely to have *Enterococcus* and more likely to have *Enhydrobacter* and *Stenotrophomonas*. No differences in abundance of *E. coli* or *Proteus* were detected.

10.1128/mSphere.00210-17.4 FIG S4  Differentially abundant bacteria at the femoral skin site in older adults who had bathed within 12 h (*n* = 35), relative to older adults who had not bathed within 12 h (*n* = 29), based on generalized linear models of abundance using the negative binomial distribution (29). Download FIG S4, TIF file, 0.2 MB. Copyright © 2017 Roghmann et al. 2017 Roghmann et al. This content is distributed under the terms of the Creative Commons Attribution 4.0 International license.

## DISCUSSION

The anterior nares, posterior pharynx and the skin over the subclavian and femoral veins are the body sites which harbor the bacteria which most commonly cause health care associated infections (7). We assessed the effect of nursing home residence on the microbiota of these body sites in older adults. Overall, we found that the OTU composition of the different body sites was similar between nursing home and community participants, but we identified differences in relative abundance. The throat microbiota was less diverse in nursing home participants; streptococci were more abundant and *Prevotella* was less abundant in nursing home participants than in community participants. Of note, there was no difference by culture- or sequence-based methods in pathogenic *Proteobacteria* in the throat. We found no differences in diversity at the anterior nares or skin sites, with the exception of the femoral skin, which was more diverse in the nursing home participants. The femoral skin was more frequently culture positive for *Enterobacteriaceae* and *P. aeruginosa* in nursing home participants. *Proteus*, *E. coli*, and *Enterococcus* were differentially more abundant in nursing home participants. Finally, we found a pattern of decreased abundance of specific *Proteobacteria* in nursing home participants at the anterior nares and both skin sites.

We anticipated that there would be differences in the microbiota of the posterior pharynx by living environment, because of culture-based differences reported by others (1–3). Although we found a trend toward decreased diversity in the nursing home participants, there was no increase in *Proteobacteria* by culture- or sequence-based methods. This could be because our sample size of nursing home participants was too small to detect a difference if one existed, or more likely, it could be because we recruited a relatively healthy group of nursing home participants, as we excluded those with antibiotic use in the past 3 months. It would be interesting to follow nursing home residents over time to assess changes in the microbiota during episodes of acute illness.

Our most abundant bacterial taxa in the throat, *Streptococcus*, *P. melaninogenica*, *R. mucilaginosa*, and *Veillonella*, were similar to what has been reported by others in healthy adults and elderly nursing home residents (8, 9). Whelan and colleagues compared the taxonomic distributions of the oropharynx of 18 elderly nursing home residents to those of healthy adults, and we found a decreased relative abundance of *Prevotella* spp. and *Veillonella* spp. The increase in *Firmicutes* such as *Streptococcus* and the decrease in *Bacteroidetes* such as *Prevotella* may be responsible for the lower diversity index. It is possible that these changes allow colonization with *Proteobacteria* more readily during periods of acute illness.

The femoral skin was also more frequently culture positive for *Enterobacteriaceae* and *P. aeruginosa* in nursing home participants than in the community participants. 16S rRNA sequences from *Proteus*, *E. coli*, and *Enterococcus* were differentially more abundant in nursing home participants than in community participants. These organisms are commonly found in the stool, which can readily contaminate the skin over the femoral vein or inguinal crease. The nursing home participants were also less likely to have bathed or showered in the past 12 h, suggesting that this finding was due to less frequent hygiene. There is currently a strong emphasis on improved skin hygiene in hospitalized patients. Bathing patients daily with chlorhexidine gluconate, an antiseptic agent with broad-spectrum activity, is an effective method for decreasing both hospital-acquired infections (i.e., bloodstream infections) and colonization with drug-resistant organisms among patients in the intensive care unit (10). Our finding supports the rationale for improved hygiene in nursing home residents to reduce the risk of infection and transmission of multidrug-resistant organisms, such as vancomyin-resistant enterococci or resistant GNR.

We also found a pattern of decreased abundance of specific *Proteobacteria* in nursing home participants at the anterior nares and both skin sites, compared with the community participants. These included *Pseudomonas*, *Burkholderia*, *Enhydrobacter*, *Stenotrophomonas*, *Achromobacter*, and *Acinetobacter*, which were unclassified at the species level. Most of these bacteria were present at a relative abundance between 1 and 10%, indicating they are potentially a significant part of the skin microbiota. Most of these taxa have been reported to cause infection in immunocompromised hosts, including people with cystic fibrosis. These may be organisms that nursing home residents are less likely to come in contact with routinely. For example, most have been reported in the water supply (11), and people who shower may have greater contact with them. Alternatively, there may be some other environmental factors related to living in a nursing home which may not support the growth of these bacteria on these skin sites. Older adults sweat less and have less oily skin, particularly in sebaceous areas such as the femoral skin. *Proteobacteria* such as *Acinetobacter*, *Stenotrophomonas*, *Pseudomonas*, and *Enhydrobacter* have been reported in the microbiota of skin sites at similar relative abundance levels (12, 13). While some members from the *Proteobacteria* phylum have been previously identified as potential contaminants in some microbiome studies (14), measures were implemented in our study to prevent such issues (e.g., inclusion of negative extraction controls and PCR-negative controls; whenever possible, UV treatment of reagents such as phosphate-buffered saline [PBS], elution buffer, and tubes; limiting the number of cycles during the PCR steps). At this point, it is premature to consider whether the presence of these bacteria is beneficial; however, a relative increase in *Proteobacteria* was also identified in the anterior nares of young adults in basic military training who did not have a skin or soft tissue infection compared to those with skin or soft tissue infection (15, 16), suggesting that this could be the case.

Our study has a few limitations. We had a smaller number of nursing home participants than community participants, which was a function of ease of recruitment. It was more difficult to recruit nursing home residents who met our eligibility criteria, in particular those without recent antibiotic use. Although we collected information on the timing of bathing or shower, we did not assess whether it was a bath or shower. A shower would expose all the skin sites to municipal water, whereas a bath, particularly in a nursing home, might be given with or without water and focus on only specific body sites (e.g., axilla and groin). Despite this, we are one of the first to assess the microbial community structure of different body sites associated with health care-associated infections, as we compared, by using genomic methods, people living in nursing homes to those who live independently in the community.

In summary, we found remarkable similarities in the bacterial communities of different body sites in older adults who lived in nursing homes compared to the people in the community, excluding from both groups people who had not been on antibiotics for the past 3 months. The taxa detected were similar, with differences in the relative abundance of specific taxa at different body sites. These shifts in abundance may facilitate colonization with pathogenic bacteria during episodes of acute illness. We also found that the femoral skin microbiota had evidence of stool contamination in the nursing home residents, providing a rationale for improved skin hygiene. Taken together, it appears that the health care environment does not alter the microbiota to the extent that antibiotics do.

## MATERIALS AND METHODS

### Study design and population.

This was a cross-sectional study of older adults from two settings: nursing homes and the community. Participants from the community were recruited from a research registry and senior centers in the Baltimore area. Participants from nursing homes were recruited from one community-based nursing home and two Veterans Administration (VA) nursing homes in Maryland. Participants from the community were 65 years of age or older and lived independently in the Baltimore area. They were excluded if any of the following were present at enrollment: BMI that was <18 or >35, chronic sinus or skin condition, immune suppression, or hospitalization or antibiotic use (including nasal or topical) within the past 3 months. Participants from NH were 65 years of age or older and lived in a participating nursing home in the Baltimore area. They had the same exclusion criteria. All participants or their legally authorized representatives gave informed consent. The study was approved by the University of Maryland, Baltimore Institutional Review Board, and the VAMHCS Research and Development Committee.

### Clinical data and specimen collection.

Potential participants were screened to document their eligibility and health status via brief survey. A medical history was taken directly from participants (older adults from the community) or their medical records (older adults from the nursing homes). Participants provided noninvasive samples from the anterior nares (nose), posterior pharynx (throat), subclavian skin, and femoral skin for culture and microbial profiling during a single study visit. Specimens were obtained separately from the anterior nares, posterior pharynx (17), and subclavian and femoral skin by using a nylon flocked swab (Copán ESwabs; Copan Diagnostics Inc., Murrieta, CA). The swab was rotated five times in each anterior nares. The tonsillar areas were wiped from side to side while rotating the swab. A 5- by 5-cm area on the skin over the right subclavian vein and right femoral vein was swabbed using firm pressure 50 times and taking at least 30 s. The swabs were placed into 1 ml of Aimes medium and the specimen medium was split, with half receiving an equal amount of RNAlater shortly after collection. Specimens with RNAlater were frozen at −80°C within 24 h of collection. Specimens for culture were worked up within 24 h of collection.

### Data management and statistical analysis of demographic and culture data.

All clinical and microbiological data were entered into study-specific centralized relational databases. Quality control was performed every 3 months via logic checks on the entirety of the databases and comparison of source documentation to the database values for 10% of the participants. The associations between dwelling and resident characteristics were measured using the chi-square test or Fisher’s exact test for categorical variables or the Wilcoxon rank sum test for continuous variables. The associations between dwelling and bacterial colonization were measured using the chi-square test or Fisher’s exact test. All statistical tests were two-tailed, and *P* values of <0.05 were considered statistically significant. These statistical analyses were conducted with Stata 12.1 software (Stata Corporation, College Station, TX).

### Microbiological methods.

Enriched samples in tryptic soy broth with 6.5% NaCl or CHROMagar Staph aureus medium (Becton Dickinson, Sparks, MD) was used for the detection of *S. aureus*. Mauve colonies growing on CHROMagar Staph aureus medium were considered positive for *S. aureus*. Any suspicious colony morphology was confirmed by Gram staining and latex agglutination (Staphaurex: Remel, Lenexa, KS) for the detection of clumping factor and protein A. Methicillin resistance was determined by using oxacillin screen agar and antibiotic susceptibilities performed following CLSI guidelines (18). GNR were enriched in brain heart infusion broth and plated onto MacConkey and Rambachrom *Acinetobacter* medium. All organisms were identified using the Vitek Compact system (BioMérieux, Durham, NC). GNR from the *Enterobacteriaceae* family and *Acinetobacter baumannii* and *Pseudomonas aeruginosa* were recorded.

### Sample processing and DNA extraction.

Total metagenomic DNA (mgDNA) was isolated as previously described (19, 20). Briefly, samples were thawed and spun down to remove the RNAlater, and the resulting cell pellet was resuspended in 1 ml ice-cold PBS and aseptically transferred into Lysing Matrix B tubes (MP Biomedicals, Solon, OH). Bacterial lysis was then performed using an enzymatic cocktail of lysozyme, mutanolysin, proteinase K, and lysostaphin, followed by mechanical lysis (bead beating). The mgDNA was then further purified using the Zymo fecal DNA kit (Zymogen). This method provides high-quality genomic DNA for bacterial community profiling via 16S rRNA gene PCR amplification and sequencing. DNA quality control/quality assurance were performed using spectrophotometric measurements with the NanoDrop system, as well as gel electrophoresis. Negative extraction controls (PBS) were processed in parallel with each extraction to ensure no contaminating DNA was introduced during the DNA extraction process and PCRs. All samples included in our analyses had negative controls.

### Microbiota profiling using 16S rRNA gene sequencing.

Microbiota profiling was performed by PCR amplification of the V3-V4 hypervariable region of the 16S rRNA gene, followed by sequencing on the Illumina HiSeq 300-bp paired-reads platform (Illumina, San Diego, CA). Sample bar coding was performed using the dual-indexing strategy for multiplexed sequencing developed at the Institute for Genome Sciences (21), which allows for up to 1,200 to 1,500 samples to be sequenced in a single HiSeq run while providing on average >50,000 read pairs per sample. Briefly, PCRs were set up in 96-well microtiter plates using the 319F (ACTCCTACGGGAGGCAGCAG) and 806R (GGACTACHVGGGTWTCTAAT) universal primers, each of which also included a linker sequence required for Illumina HiSeq 300 bp paired-ends sequencing, and also a 12-bp heterogeneity spacer index sequence aimed at minimizing biases associated with low-diversity amplicon sequencing (21). First-step PCR amplifications were performed using the Phusion high-fidelity PCR master mix (Thermo Fisher, USA) and 9 μl of extracted DNA as the template in a total reaction volume of 25 μl (22). Reactions were run in a DNA Engine Tetrad 2 thermocycler (Bio-Rad, USA) using the following cycling parameters: 3 min at 95°C, followed by 30 cycles of 30 s at 95°C, 30 s at 58°C, and 1 min at 72°C, with a final step of 5 min at 72°C. A 1:20 dilution of the step one products was performed prior to step two amplification. Second-step PCRs were set up in 96-well microtiter plates using custom bar code primers unique for each sample along with Phusion high-fidelity PCR master mix (Thermo Fisher, USA) and 1 µl of diluted first-step product. Cycling parameters for second-step PCR were 30 s at 95°C, followed by 10 cycles of 30 s at 95°C, 30 s at 58°C, and 1 min at 72°C, with a final step of 5 min at 72°C. No-template negative controls were processed for each primer pair. The presence of PCR amplicons was confirmed using gel electrophoresis, after which the SequalPrep normalization plate kit (Life Technologies, Inc.) was used for cleanup and normalization (25 ng of 16S PCR amplicon pooled for each sample) before sequencing.

### Data processing and statistical analyses of microbiome data.

Following sequencing, 16S rRNA reads were initially screened for low-quality bases and short read lengths (21). Paired-end read pairs were then assembled using the PANDAseq software (23), and the resulting consensus sequences were demultiplexed (i.e., assigned to their original sample), trimmed of bar codes and primers, and assessed for chimeras using UCHIME (24) in *de novo* mode implemented in QIIME (v. 1.9.1) (25). Quality-trimmed sequences were then clustered *de novo* into operational taxonomic units at 97% similarity cutoff using QIIME, and taxonomic assignments were performed using the RDP classifier implemented in QIIME and the Greengenes database (v. 13.8) database as a reference.

The resulting taxonomic assignments were imported as a BIOM-formatted file into R (v. 3.3.2) using RStudio (v. 1.0.44) integrated development environment (IDE), and processed/analyzed using the following R packages: Phyloseq (v. 1.19.1) (26), Vegan (v. 2.4-1) (30), and gpplot2 (v. 2.2.1). When appropriate, taxonomic assignment data were normalized to account for uneven sampling depth with metagenomeSeq’s cumulative sum scaling (CSS; implemented in R) (27), a novel normalization method that has been shown to be less biased than the standard approach (total sum normalization). Good’s coverage index was calculated using QIIME for each sample in order to ensure appropriate sequence coverage: samples with Good’s coverage of <0.9 were discarded from the analyses. In addition, ultralow abundant and likely to be spurious OTUs (<0.005% relative abundance and present in <10% of samples) were removed from the OTU table prior to the analyses described below.

Before normalization, within-sample comparisons using alpha diversity measures were performed with the observed and Chao1 estimators, as well as the Shannon diversity index, calculated using the Phyloseq R package. Because alpha diversity metrics can be susceptible to uneven sampling depth between samples, alpha diversity measures were compared after rarefaction to the minimum sampling depth of 500 sequences. The associations between participant dwelling and alpha diversity data were measured using the Wilcoxon rank sum test.

Beta diversity (between-sample) comparisons were performed from CSS-normalized data through principal-component analysis (PCoA) plots of Bray-Curtis distances determined using QIIME and tested for significance using the ANOSIM algorithm (9,999 permutations) (28) implemented in the Vegan package in R.

Determination of statistically significant differences (*P* < 0.05) for OTU bacterial relative abundance levels between the CB and NH cohorts was performed using DESeq2 (29) implemented in R, which utilizes the Benjamini-Hochberg multiple-inference correction. DESeq2 was used due to its high power in computing statistical significance of differentially abundant features in high-dimensional data sets derived from relatively small sample sizes. Differentially abundant OTUs were selected with an adjusted *P* value of <0.01.

### Accession number(s).

Sequence data generated in this study were deposited with GenBank and linked to BioProject number PRJNA388722 in the NCBI BioProject database.
