# Supplementary figures and images for: Comparison of the Microbiota of Older Adults Living in Nursing Homes and the Community
Source: mSphere. 2017 Sep 13;2(5):e00210-17. doi: 10.1128/mSphere.00210-17 (PMC5597966; doi:10.1128/mSphere.00210-17)

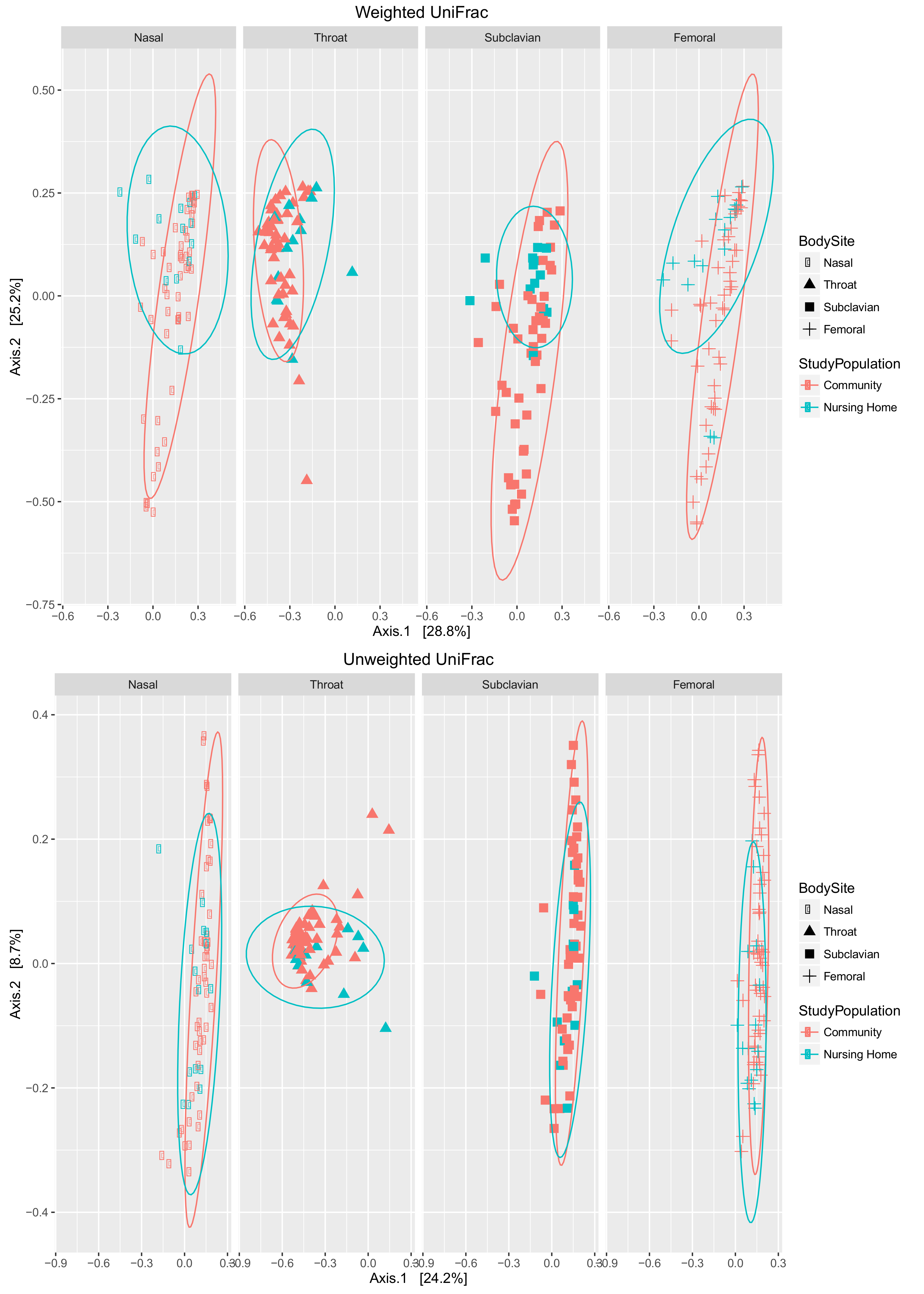

Supplement: FIG S2 [file sph005172361sf2.tif]

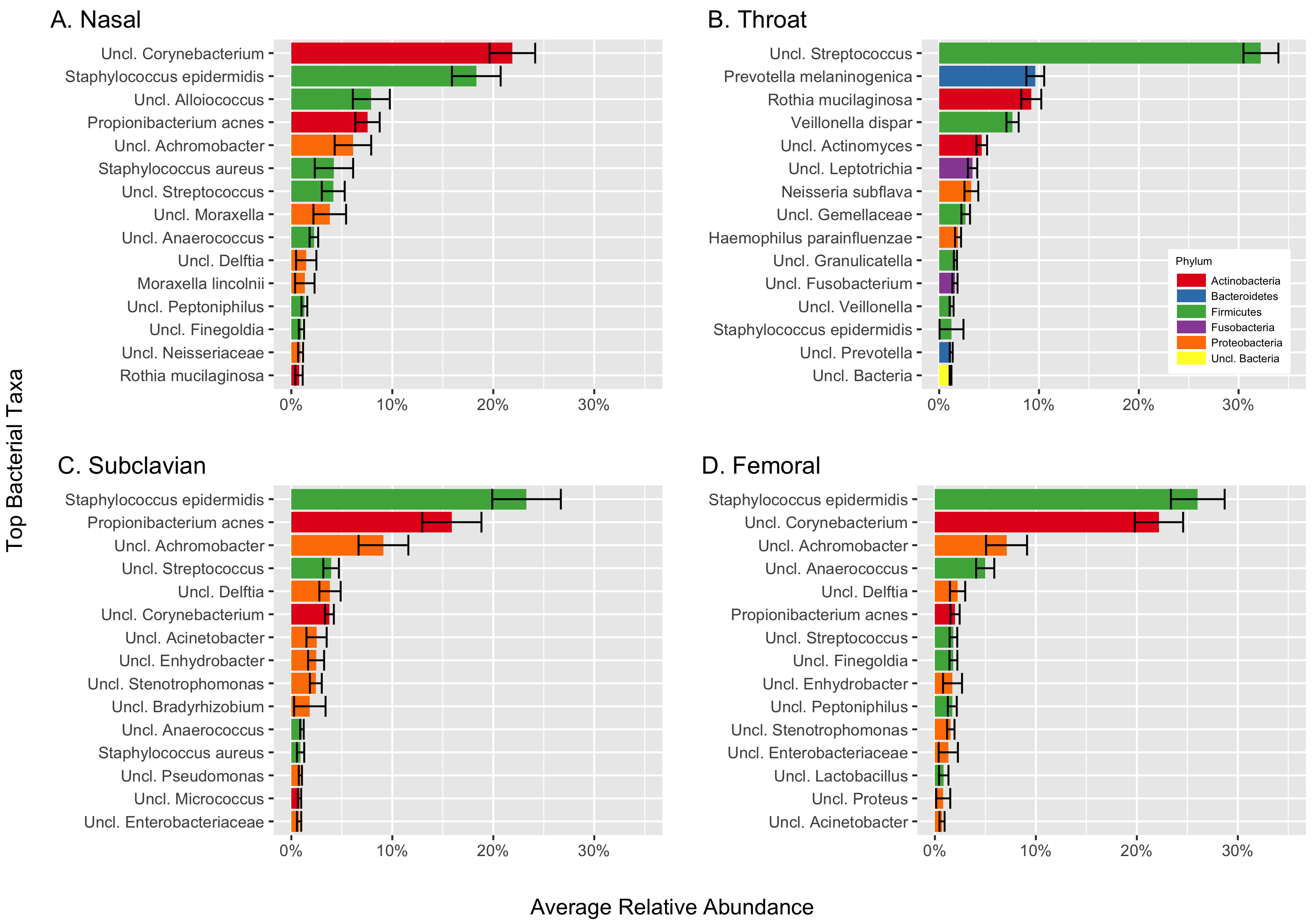

Supplement: FIG S3 [file sph005172361sf3.tif]

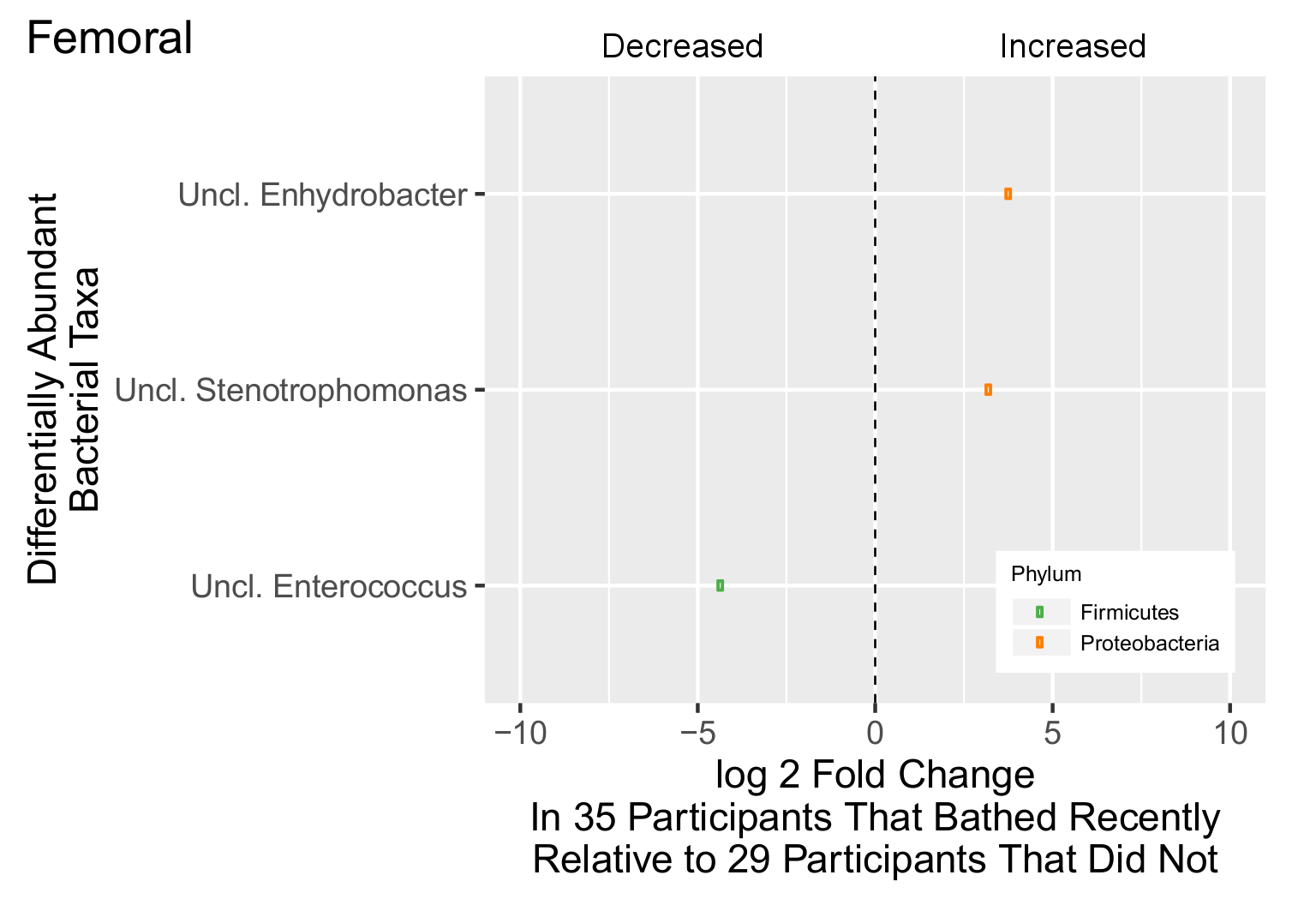

Supplement: FIG S4 [file sph005172361sf4.tif]
